# *N*-Benzyl­aniline

**DOI:** 10.1107/S1600536811014553

**Published:** 2011-04-22

**Authors:** Richard Betz, Cedric McCleland, Harold Marchand

**Affiliations:** a Nelson Mandela Metropolitan University, Summerstrand Campus, Department of Chemistry, University Way, Summerstrand, PO Box 77000, Port Elizabeth 6031, South Africa

## Abstract

The title compound, C_13_H_13_N, is an *N*-alkyl­ated derivative of aniline. The N atom is present in a nearly planar mol­ecular geometry (angles sums at the N atom are 358 and 359° in the two molecules of the asymmetric unit). The planes defined by the aromatic rings intersect at angles of 80.76 (4) and 81.40 (4)° in the two molecules. In the crystal, N—H⋯*Cg* inter­actions connect the two mol­ecules of the asymmetric unit to form infinite homodromic chains along the crystallographic *b* axis [N⋯π = 3.4782 (12) and 3.4642 (13) Å].

## Related literature

For the crystal structure analysis of a ruthenium coordination compound featuring the title compound as a ligand, see: Casey *et al.* (2006 ▶). For the crystal structure analysis of a rhodium coordination compound containing the title compound as a ligand, see: Marcazzan *et al.* (2003 ▶).
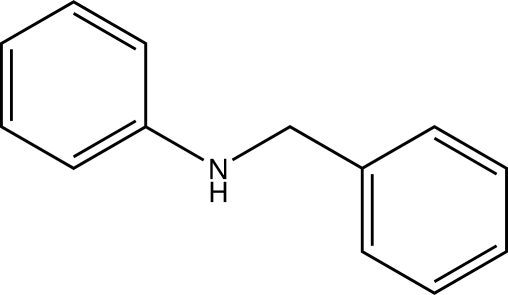


## Experimental

### 

#### Crystal data

C_13_H_13_N
*M* *_r_* = 183.24
Monoclinic, 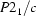

*a* = 18.8185 (6) Å
*b* = 5.7911 (2) Å
*c* = 19.3911 (7) Å
β = 103.338 (1)°
*V* = 2056.24 (12) Å^3^
*Z* = 8
Mo *K*α radiation
μ = 0.07 mm^−1^
*T* = 200 K
0.60 × 0.33 × 0.13 mm

#### Data collection

Bruker APEXII CCD diffractometer
18958 measured reflections
4929 independent reflections
4088 reflections with *I* > 2σ(*I*)
*R*_int_ = 0.038

#### Refinement

*R*[*F* ^2^ > 2σ(*F* ^2^)] = 0.044
*wR*(*F* ^2^) = 0.118
*S* = 1.04
4929 reflections
261 parameters
H atoms treated by a mixture of independent and constrained refinement
Δρ_max_ = 0.17 e Å^−3^
Δρ_min_ = −0.15 e Å^−3^

### 

Data collection: *APEX2* (Bruker, 2010 ▶); cell refinement: *SAINT* (Bruker, 2010 ▶); data reduction: *SAINT*; program(s) used to solve structure: *SHELXS97* (Sheldrick, 2008 ▶); program(s) used to refine structure: *SHELXL97* (Sheldrick, 2008 ▶); molecular graphics: *ORTEP-3* (Farrugia, 1997 ▶) and *Mercury* (Macrae *et al.*, 2006 ▶); software used to prepare material for publication: *SHELXL97* and *PLATON* (Spek, 2009 ▶).

## Supplementary Material

Crystal structure: contains datablocks I, global. DOI: 10.1107/S1600536811014553/ds2106sup1.cif

Supplementary material file. DOI: 10.1107/S1600536811014553/ds2106Isup2.cdx

Structure factors: contains datablocks I. DOI: 10.1107/S1600536811014553/ds2106Isup3.hkl

Additional supplementary materials:  crystallographic information; 3D view; checkCIF report

## Figures and Tables

**Table 1:** Hydrogen-bond geometry (Å, °) *Cg*1 is the centroid of the C21–C26 ring and *Cg*2 is the centroid of the C41–C46 ring.

*D*—H⋯*A*	*D*—H	H⋯*A*	*D*⋯*A*	*D*—H⋯*A*
N1—H71⋯*Cg*1^i^	0.889 (17)	2.608 (17)	3.4782 (12)	166.0 (14)
N2—H72⋯*Cg*2^i^	0.858 (17)	2.625 (17)	3.4642 (13)	165.5 (15)

Symmetry code: (i) 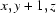.

**Fig. 1.**
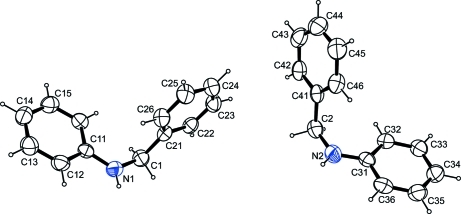
The asymmetric unit of the title compound, with atom labels and anisotropic displacement ellipsoids (drawn at 50% probability level).

**Fig. 2.**
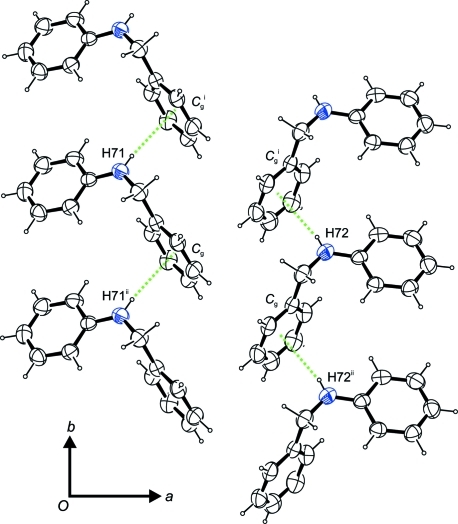
Intermolecular N–H···Cg contacts, viewed along [00\=1]. Symmetry operators: ix, y + 1, z; iix, y - 1, z.

**Fig. 3.**
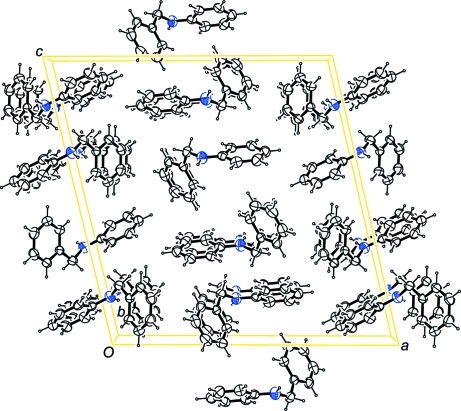
Molecular packing of the title compound, viewed along [010] (anisotropic displacement ellipsoids drawn at 50% probability level).

## supplementary crystallographic information

### Comment

The coordination behaviour of monodentate ligands is influenced by electronic as well as steric factors. In this aspect, derivatives of aniline are particularily interesting and promising compounds due to a series of reasons: first, they can act as neutral or – upon deprotonation – as anionic ligands. Second, the derivatization of the aromatic system of aniline allows for the fine-tuning of the basicity and nucleophilicity of the N atom and thus its coordination behaviour in terms of Lewis basicity. Third, the steric pretense of the ligand can be varied by applying different patterns of substituents among the aromatic regime as well as by endowing the N atom itself with sterically more demanding groups. In our continuous efforts to elucidate the coordination behaviour of *N* donor ligands, it seemed necessary to determine the crystal structure of the title compound to enable comparative studies with the coordination compounds obtained. So far, only two structure determinations involving the title compound as a ligand are present in the literature (Casey *et al.*, 2006; Marcazzan *et al.*, 2003).

The molecular geometry around both molecules of the asymmetric unit is essentially planar with X—N—Y angles ranging from 117.0 (10)° to 124.54 (11)°. The biggest of these angles in the title compound is found for both molecules for the C—N—C angle. The phenyl rings within one molecule of the asymmetric unit are nearly orientated perpendicular to each other, the least-squares planes defined by the aromatic rings within one molecule enclose angles of 80.76 (4)° and 81.40 (4)°, respectively (Fig. 1).

The N—H groups do not interact with each other. Instead, the formation of N—H···*Cg* contacts is observed in the crystal structure. These contacts exclusively use the aromatic moiety of the benzyl substituent as acceptor and are present only between one of the molecules of the asymmetric unit and its translation symmetry-generated equivalents (Fig. 2). In total, the formation of two one-dimensional chains of molecules along the crystallographic *b* axis is observed.

The packing of the title compound is shown in Fig. 3.

### Experimental

The compound was obtained commercially (Aldrich). Crystals suitable for the X-ray diffraction study were taken directly from the provided compound.

### Refinement

Carbon-bound H atoms were placed in calculated positions (C—H 0.95 Å for aromatic C atoms, C—H 0.99 Å for aliphatic C atoms) and were included in the refinement in the riding model approximation, with *U*(H) set to 1.2*U*_eq_(C). The nitrogen-bound H atoms were located on a difference Fourier map and refined freely.

### Crystal data

**Table:**

C_13_H_13_N	*F*(000) = 784
*M**_r_* = 183.24	*D*_x_ = 1.184 Mg m^−^^3^
Monoclinic, *P*2_1_/*c*	Mo *K*α radiation, λ = 0.71073 Å
Hall symbol: -P 2ybc	Cell parameters from 9996 reflections
*a* = 18.8185 (6) Å	θ = 2.2–28.3°
*b* = 5.7911 (2) Å	µ = 0.07 mm^−^^1^
*c* = 19.3911 (7) Å	*T* = 200 K
β = 103.338 (1)°	Block, colourless
*V* = 2056.24 (12) Å^3^	0.60 × 0.33 × 0.13 mm
*Z* = 8	

### Data collection

**Table:**

Bruker APEXII CCD diffractometer	4088 reflections with *I* > 2σ(*I*)
Radiation source: fine-focus sealed tube	*R*_int_ = 0.038
graphite	θ_max_ = 28.0°, θ_min_ = 1.1°
φ and ω scans	*h* = −24→24
18958 measured reflections	*k* = −7→7
4929 independent reflections	*l* = −24→25

### Refinement

**Table:**

Refinement on *F*^2^	Primary atom site location: structure-invariant direct methods
Least-squares matrix: full	Secondary atom site location: difference Fourier map
*R*[*F*^2^ > 2σ(*F*^2^)] = 0.044	Hydrogen site location: inferred from neighbouring sites
*wR*(*F*^2^) = 0.118	H atoms treated by a mixture of independent and constrained refinement
*S* = 1.04	*w* = 1/[σ^2^(*F*_o_^2^) + (0.0511*P*)^2^ + 0.4031*P*] where *P* = (*F*_o_^2^ + 2*F*_c_^2^)/3
4929 reflections	(Δ/σ)_max_ < 0.001
261 parameters	Δρ_max_ = 0.17 e Å^−^^3^
0 restraints	Δρ_min_ = −0.15 e Å^−^^3^

### Fractional atomic coordinates and isotropic or equivalent isotropic displacement parameters (Å^2^)

**Table:**

	*x*	*y*	*z*	*U*_iso_*/*U*_eq_	
N1	0.03311 (6)	0.97897 (19)	0.16847 (6)	0.0493 (2)	
H71	0.0550 (9)	1.099 (3)	0.1535 (9)	0.070 (5)*	
N2	0.46883 (6)	0.44222 (19)	0.15115 (6)	0.0540 (3)	
H72	0.4494 (9)	0.561 (3)	0.1275 (9)	0.068 (5)*	
C1	0.07935 (7)	0.8108 (2)	0.21148 (6)	0.0479 (3)	
H1A	0.0513	0.7377	0.2430	0.058*	
H1B	0.1209	0.8935	0.2421	0.058*	
C2	0.42036 (7)	0.2780 (2)	0.17124 (7)	0.0503 (3)	
H2A	0.3776	0.3629	0.1800	0.060*	
H2B	0.4458	0.2073	0.2167	0.060*	
C11	−0.03932 (6)	0.94047 (18)	0.13574 (6)	0.0385 (2)	
C12	−0.07788 (7)	1.1118 (2)	0.09194 (7)	0.0493 (3)	
H12	−0.0535	1.2488	0.0835	0.059*	
C13	−0.15091 (7)	1.0841 (2)	0.06090 (7)	0.0560 (3)	
H13	−0.1763	1.2029	0.0314	0.067*	
C14	−0.18771 (7)	0.8866 (2)	0.07191 (7)	0.0529 (3)	
H14	−0.2382	0.8690	0.0508	0.063*	
C15	−0.14974 (7)	0.7155 (2)	0.11411 (7)	0.0501 (3)	
H15	−0.1744	0.5781	0.1217	0.060*	
C16	−0.07616 (6)	0.7395 (2)	0.14580 (6)	0.0436 (3)	
H16	−0.0509	0.6186	0.1744	0.052*	
C21	0.10986 (5)	0.62091 (19)	0.17312 (6)	0.0386 (2)	
C22	0.15966 (6)	0.4669 (2)	0.21269 (6)	0.0442 (3)	
H22	0.1728	0.4813	0.2628	0.053*	
C23	0.19021 (7)	0.2936 (2)	0.18036 (7)	0.0522 (3)	
H23	0.2249	0.1917	0.2081	0.063*	
C24	0.17031 (7)	0.2678 (2)	0.10746 (7)	0.0546 (3)	
H24	0.1910	0.1478	0.0850	0.066*	
C25	0.12036 (7)	0.4171 (2)	0.06781 (7)	0.0536 (3)	
H25	0.1062	0.3987	0.0178	0.064*	
C26	0.09050 (6)	0.5938 (2)	0.09998 (6)	0.0464 (3)	
H26	0.0566	0.6972	0.0719	0.056*	
C31	0.54218 (6)	0.40591 (19)	0.15682 (6)	0.0412 (2)	
C32	0.57707 (6)	0.2029 (2)	0.18503 (6)	0.0431 (3)	
H32	0.5496	0.0818	0.1994	0.052*	
C33	0.65154 (7)	0.1774 (2)	0.19213 (6)	0.0489 (3)	
H33	0.6747	0.0386	0.2116	0.059*	
C34	0.69271 (7)	0.3490 (2)	0.17147 (7)	0.0545 (3)	
H34	0.7438	0.3300	0.1766	0.065*	
C35	0.65833 (8)	0.5495 (2)	0.14311 (8)	0.0568 (3)	
H35	0.6861	0.6695	0.1287	0.068*	
C36	0.58427 (7)	0.5778 (2)	0.13539 (7)	0.0508 (3)	
H36	0.5616	0.7163	0.1152	0.061*	
C41	0.39244 (6)	0.0851 (2)	0.11933 (6)	0.0414 (2)	
C42	0.34291 (6)	−0.0710 (2)	0.13551 (7)	0.0495 (3)	
H42	0.3284	−0.0566	0.1791	0.059*	
C43	0.31429 (7)	−0.2472 (3)	0.08936 (8)	0.0606 (4)	
H43	0.2796	−0.3509	0.1008	0.073*	
C44	0.33613 (8)	−0.2722 (3)	0.02670 (8)	0.0645 (4)	
H44	0.3169	−0.3940	−0.0050	0.077*	
C45	0.38576 (7)	−0.1206 (3)	0.01009 (7)	0.0620 (4)	
H45	0.4011	−0.1385	−0.0330	0.074*	
C46	0.41358 (7)	0.0583 (2)	0.05581 (6)	0.0506 (3)	
H46	0.4474	0.1635	0.0436	0.061*	

### Atomic displacement parameters (Å^2^)

**Table:**

	*U*^11^	*U*^22^	*U*^33^	*U*^12^	*U*^13^	*U*^23^
N1	0.0465 (5)	0.0416 (5)	0.0569 (6)	−0.0028 (4)	0.0063 (5)	0.0007 (5)
N2	0.0498 (6)	0.0445 (6)	0.0672 (7)	0.0091 (5)	0.0125 (5)	0.0067 (5)
C1	0.0479 (6)	0.0532 (7)	0.0393 (6)	0.0025 (5)	0.0030 (5)	−0.0033 (5)
C2	0.0487 (6)	0.0567 (7)	0.0475 (6)	0.0072 (5)	0.0154 (5)	−0.0017 (5)
C11	0.0435 (5)	0.0363 (5)	0.0368 (5)	0.0012 (4)	0.0117 (4)	−0.0048 (4)
C12	0.0557 (7)	0.0366 (6)	0.0536 (7)	−0.0013 (5)	0.0086 (5)	0.0023 (5)
C13	0.0558 (7)	0.0511 (7)	0.0559 (7)	0.0079 (6)	0.0020 (6)	0.0040 (6)
C14	0.0422 (6)	0.0648 (8)	0.0511 (7)	−0.0004 (6)	0.0096 (5)	−0.0066 (6)
C15	0.0502 (6)	0.0515 (7)	0.0535 (7)	−0.0084 (5)	0.0223 (5)	−0.0027 (6)
C16	0.0492 (6)	0.0408 (6)	0.0438 (6)	0.0015 (5)	0.0168 (5)	0.0043 (5)
C21	0.0346 (5)	0.0438 (6)	0.0367 (5)	−0.0047 (4)	0.0071 (4)	0.0022 (4)
C22	0.0397 (5)	0.0501 (6)	0.0399 (5)	−0.0035 (5)	0.0028 (4)	0.0052 (5)
C23	0.0435 (6)	0.0506 (7)	0.0597 (7)	0.0057 (5)	0.0062 (5)	0.0080 (6)
C24	0.0498 (7)	0.0556 (7)	0.0614 (8)	0.0044 (6)	0.0190 (6)	−0.0047 (6)
C25	0.0536 (7)	0.0687 (8)	0.0400 (6)	0.0030 (6)	0.0143 (5)	−0.0040 (6)
C26	0.0459 (6)	0.0560 (7)	0.0363 (5)	0.0054 (5)	0.0074 (5)	0.0049 (5)
C31	0.0474 (6)	0.0377 (5)	0.0370 (5)	0.0026 (4)	0.0070 (4)	−0.0055 (4)
C32	0.0479 (6)	0.0394 (6)	0.0401 (5)	−0.0001 (5)	0.0061 (5)	0.0004 (5)
C33	0.0486 (6)	0.0484 (7)	0.0452 (6)	0.0062 (5)	0.0017 (5)	−0.0018 (5)
C34	0.0458 (6)	0.0604 (8)	0.0560 (7)	−0.0031 (6)	0.0092 (5)	−0.0094 (6)
C35	0.0614 (8)	0.0492 (7)	0.0636 (8)	−0.0109 (6)	0.0222 (6)	−0.0061 (6)
C36	0.0644 (8)	0.0354 (6)	0.0535 (7)	0.0016 (5)	0.0154 (6)	−0.0007 (5)
C41	0.0346 (5)	0.0499 (6)	0.0393 (5)	0.0108 (4)	0.0078 (4)	0.0067 (5)
C42	0.0399 (6)	0.0585 (7)	0.0512 (6)	0.0106 (5)	0.0130 (5)	0.0139 (6)
C43	0.0415 (6)	0.0577 (8)	0.0776 (9)	0.0008 (6)	0.0037 (6)	0.0134 (7)
C44	0.0528 (7)	0.0674 (9)	0.0631 (8)	0.0030 (7)	−0.0074 (6)	−0.0091 (7)
C45	0.0552 (7)	0.0858 (10)	0.0419 (6)	0.0070 (7)	0.0047 (6)	−0.0080 (7)
C46	0.0442 (6)	0.0679 (8)	0.0403 (6)	0.0015 (6)	0.0112 (5)	0.0028 (6)

### Geometric parameters (Å, °)

**Table:**

N1—C11	1.3823 (14)	C23—H23	0.9500
N1—C1	1.4375 (15)	C24—C25	1.3743 (18)
N1—H71	0.889 (17)	C24—H24	0.9500
N2—C31	1.3753 (15)	C25—C26	1.3837 (18)
N2—C2	1.4327 (17)	C25—H25	0.9500
N2—H72	0.858 (17)	C26—H26	0.9500
C1—C21	1.5131 (16)	C31—C36	1.3940 (17)
C1—H1A	0.9900	C31—C32	1.3953 (15)
C1—H1B	0.9900	C32—C33	1.3840 (16)
C2—C41	1.5141 (17)	C32—H32	0.9500
C2—H2A	0.9900	C33—C34	1.3755 (19)
C2—H2B	0.9900	C33—H33	0.9500
C11—C16	1.3915 (15)	C34—C35	1.380 (2)
C11—C12	1.3955 (16)	C34—H34	0.9500
C12—C13	1.3766 (18)	C35—C36	1.3768 (19)
C12—H12	0.9500	C35—H35	0.9500
C13—C14	1.3792 (19)	C36—H36	0.9500
C13—H13	0.9500	C41—C42	1.3853 (17)
C14—C15	1.3757 (19)	C41—C46	1.3877 (16)
C14—H14	0.9500	C42—C43	1.382 (2)
C15—C16	1.3863 (17)	C42—H42	0.9500
C15—H15	0.9500	C43—C44	1.377 (2)
C16—H16	0.9500	C43—H43	0.9500
C21—C26	1.3894 (15)	C44—C45	1.373 (2)
C21—C22	1.3895 (15)	C44—H44	0.9500
C22—C23	1.3774 (18)	C45—C46	1.3852 (19)
C22—H22	0.9500	C45—H45	0.9500
C23—C24	1.3844 (19)	C46—H46	0.9500
			
C11—N1—C1	123.90 (10)	C25—C24—C23	119.49 (12)
C11—N1—H71	117.2 (10)	C25—C24—H24	120.3
C1—N1—H71	117.0 (10)	C23—C24—H24	120.3
C31—N2—C2	124.54 (11)	C24—C25—C26	120.64 (11)
C31—N2—H72	117.4 (11)	C24—C25—H25	119.7
C2—N2—H72	117.1 (11)	C26—C25—H25	119.7
N1—C1—C21	117.05 (10)	C25—C26—C21	120.37 (11)
N1—C1—H1A	108.0	C25—C26—H26	119.8
C21—C1—H1A	108.0	C21—C26—H26	119.8
N1—C1—H1B	108.0	N2—C31—C36	119.72 (11)
C21—C1—H1B	108.0	N2—C31—C32	122.21 (11)
H1A—C1—H1B	107.3	C36—C31—C32	118.05 (11)
N2—C2—C41	117.01 (10)	C33—C32—C31	120.21 (11)
N2—C2—H2A	108.0	C33—C32—H32	119.9
C41—C2—H2A	108.0	C31—C32—H32	119.9
N2—C2—H2B	108.0	C34—C33—C32	121.28 (12)
C41—C2—H2B	108.0	C34—C33—H33	119.4
H2A—C2—H2B	107.3	C32—C33—H33	119.4
N1—C11—C16	122.75 (10)	C33—C34—C35	118.71 (12)
N1—C11—C12	118.95 (10)	C33—C34—H34	120.6
C16—C11—C12	118.29 (11)	C35—C34—H34	120.6
C13—C12—C11	120.67 (11)	C36—C35—C34	120.88 (12)
C13—C12—H12	119.7	C36—C35—H35	119.6
C11—C12—H12	119.7	C34—C35—H35	119.6
C12—C13—C14	120.99 (12)	C35—C36—C31	120.86 (12)
C12—C13—H13	119.5	C35—C36—H36	119.6
C14—C13—H13	119.5	C31—C36—H36	119.6
C15—C14—C13	118.63 (12)	C42—C41—C46	118.27 (12)
C15—C14—H14	120.7	C42—C41—C2	118.63 (10)
C13—C14—H14	120.7	C46—C41—C2	123.09 (11)
C14—C15—C16	121.34 (11)	C43—C42—C41	121.15 (12)
C14—C15—H15	119.3	C43—C42—H42	119.4
C16—C15—H15	119.3	C41—C42—H42	119.4
C15—C16—C11	120.05 (11)	C44—C43—C42	119.85 (13)
C15—C16—H16	120.0	C44—C43—H43	120.1
C11—C16—H16	120.0	C42—C43—H43	120.1
C26—C21—C22	118.41 (11)	C45—C44—C43	119.83 (14)
C26—C21—C1	122.95 (10)	C45—C44—H44	120.1
C22—C21—C1	118.63 (10)	C43—C44—H44	120.1
C23—C22—C21	121.03 (11)	C44—C45—C46	120.34 (13)
C23—C22—H22	119.5	C44—C45—H45	119.8
C21—C22—H22	119.5	C46—C45—H45	119.8
C22—C23—C24	120.03 (11)	C45—C46—C41	120.55 (12)
C22—C23—H23	120.0	C45—C46—H46	119.7
C24—C23—H23	120.0	C41—C46—H46	119.7
			
C11—N1—C1—C21	78.24 (15)	C1—C21—C26—C25	179.85 (11)
C31—N2—C2—C41	−79.87 (15)	C2—N2—C31—C36	179.76 (11)
C1—N1—C11—C16	5.02 (17)	C2—N2—C31—C32	−1.69 (18)
C1—N1—C11—C12	−176.44 (11)	N2—C31—C32—C33	−177.61 (11)
N1—C11—C12—C13	−177.14 (12)	C36—C31—C32—C33	0.96 (16)
C16—C11—C12—C13	1.47 (18)	C31—C32—C33—C34	−0.32 (18)
C11—C12—C13—C14	−0.3 (2)	C32—C33—C34—C35	−0.12 (19)
C12—C13—C14—C15	−0.7 (2)	C33—C34—C35—C36	−0.1 (2)
C13—C14—C15—C16	0.59 (19)	C34—C35—C36—C31	0.8 (2)
C14—C15—C16—C11	0.56 (18)	N2—C31—C36—C35	177.41 (12)
N1—C11—C16—C15	176.99 (11)	C32—C31—C36—C35	−1.20 (17)
C12—C11—C16—C15	−1.57 (16)	N2—C2—C41—C42	−177.15 (10)
N1—C1—C21—C26	−5.22 (17)	N2—C2—C41—C46	2.21 (17)
N1—C1—C21—C22	174.60 (10)	C46—C41—C42—C43	−0.91 (17)
C26—C21—C22—C23	1.09 (17)	C2—C41—C42—C43	178.48 (11)
C1—C21—C22—C23	−178.73 (11)	C41—C42—C43—C44	1.26 (18)
C21—C22—C23—C24	−1.32 (18)	C42—C43—C44—C45	−0.6 (2)
C22—C23—C24—C25	0.4 (2)	C43—C44—C45—C46	−0.5 (2)
C23—C24—C25—C26	0.7 (2)	C44—C45—C46—C41	0.8 (2)
C24—C25—C26—C21	−0.9 (2)	C42—C41—C46—C45	−0.13 (17)
C22—C21—C26—C25	0.04 (17)	C2—C41—C46—C45	−179.48 (11)

### Hydrogen-bond geometry (Å, °)

**Table:**

*D*—H···*A*	*D*—H	H···*A*	*D*···*A*	*D*—H···*A*
N1—H71···*Cg*1^i^	0.889 (17)	2.608 (17)	3.4782 (12)	166.0 (14)
N2—H72···*Cg*2^i^	0.858 (17)	2.625 (17)	3.4642 (13)	165.5 (15)

Symmetry codes: (i) *x*, *y*+1, *z*.
